# A hop testing alternative for functional performance following anterior cruciate ligament reconstruction

**DOI:** 10.1371/journal.pone.0309003

**Published:** 2024-08-16

**Authors:** Zachary Ripic, Michael Letter, Brandon Schoenwether, Lee D. Kaplan, Michael G. Baraga, Joseph P. Costello II, Jordan Eskenazi, Molly Dennison, Thomas M. Best, Joseph F. Signorile, Moataz Eltoukhy

**Affiliations:** 1 Department of Kinesiology and Sport Sciences, University of Miami, Coral Gables, FL, United States of America; 2 Department of Orthopaedics, University of Miami Health System ‐ Sports Medicine Institute, Coral Gables, FL, United States of America; 3 Miller School of Medicine, University of Miami, Miami, FL, United States of America; 4 Department of Industrial and Systems Engineering, University of Miami, Coral Gables, FL, United States of America; 5 Department of Physical Therapy, University of Miami Miller School of Medicine, Miami, FL, United States of America; IRCCS Istituto Ortopedico Rizzoli, ITALY

## Abstract

The purpose of this work was to provide a simple method to determine reactive strength during the 6-meter timed hop test (6mTH) and evaluate its association with isokinetic peak torque in patients following anterior cruciate ligament reconstruction (ACLR). Twenty-nine ACLR patients who were at least four months from surgery were included in this analysis. Participants were brought into the laboratory on one occasion to complete functional testing. Quadriceps and hamstring isokinetic testing was completed bilaterally at 60, 180, and 300 deg∙s^-1^, using extension peak torque from each speed as the outcome measure. The 6mTH was completed bilaterally using a marker-based motion capture system, and reactive strength ratio (RSR) was calculated from the vertical velocity of the pelvis during the test. An adjustment in RSR was made using the velocity of the 6mTH test to account for different strategies employed across participants. Repeated measures correlations were used to determine associations among isokinetic and hop testing variables. A two-way mixed analysis of variance was used to determine differences in isokinetic and hop testing variables between operated and non-operated legs and across male and female participants. Moderate positive associations were found between RSR (and adjusted RSR) and isokinetic peak torque at all speeds (*r* = .527 to .577). Mean comparisons showed significant main effects for leg and sex. Patients showed significant deficits in their operated versus non-operated legs in all isokinetic and hop testing variables, yet only isokinetic peak torque and timed hop time showed significant differences across male and female groups. Preliminary results are promising but further development is needed to validate other accessible technologies available to calculate reactive strength during functional testing after ACLR. Pending these developments, the effects of movement strategies, demographics, and levels of participation on RSR can then be explored to translate this simple method to clinical environments.

## Introduction

Several studies have maintained that a criterion-based progression should be used for return to sport (RTS) following anterior cruciate ligament reconstruction (ACLR) rather than the traditional “time since surgery” approach [1–4]. As noted by Andrade et al. (2020), clinical practice guidelines recommend that clinicians use graft biological healing, clinical examinations and surveys, strength and functional testing, and psychological readiness milestones to advance the athlete through the rehabilitation process [3–7]. When examining readiness to return to unrestricted sport, 52.5% of surveyed surgeons indicated functional testing scores were the most important factor to determine readiness, while 37.5% and 5.0% indicated time since surgery or muscle strength, respectively [8]. In a systematic review by Roe et al. 2021 [9], the top three testing criteria for RTS were single leg hop for distance (87.3%), triple hop (61.9%), and crossover hop (47.6%). Common strength testing modalities were isokinetic testing at 60 degrees∙s^-1^ (33.3%) and 180 degrees∙s^-1^ (25.4%), followed by isometric testing at 90 degrees of knee flexion (23.8%). Furthermore, a survey of 1074 physical therapists indicated that a large (>80%) majority use strength and functional test criteria to progress patients to jogging and modified sports activity; yet many use manual muscle testing in their progression determination, and over half of respondents indicated that they do not require additional testing to progress patients to later stages of rehabilitation [10]. Others have recommended incorporating biomechanical measures into functional testing [11, 12]; and 84.8% of surgeons reported quantitative assessment technologies would improve outcomes of ACLR recovery, yet only 43.5% currently have the capacity to do so due to limitations including equipment expense, time needed for testing, lack of training, lack of testing protocols, and lack of validation of technologies [8].

Although hop tests are common in RTS testing, there are several drawbacks that limit their effectiveness for identifying underlying functional deficits. The common use of limb symmetry index (LSI), defined as the operative limb performance expressed as percentage of the non-operative limb, masks performance deficits [13, 14]. Hop distance LSI levels have been shown to be within normal levels, despite a shift in negative joint work distribution during single leg hop testing [15, 16]. Given the frequent recommendations for regular objective testing [1], hop tests remain an accessible approach to obtain numerical values for longitudinal monitoring [17], whereas other recommendations like fixed dynamometry isokinetic strength testing may prove difficult due to time or equipment limitations in clinical settings. Therefore, it is clear that there is a pressing need to provide consequential biomechanical measures that can augment traditional hop testing [12] and improve outcomes after ACLR [8].

Besides traditional ACLR RTS hop testing, the reactive strength index (RSI), the ratio between jump height and ground contact time, has received much attention as a performance indicator in different athletic groups [18]. Moderate associations between maximum- and reactive-strength have been found in collegiate athletes, where stronger athletes show higher RSIs during drop vertical jumps [18, 19]. Others have found moderate associations of RSI with acceleration speed and top speed; as well as a strong association with change of direction speed [18, 20]. Females have shown lower RSI compared to male participants during countermovement jump testing [21]; and, while male athletes have shown higher RSIs, statistically significant differences could be sport dependent across collegiate programs [21, 22].

Two studies have investigated measures of stretch-shortening cycle capacity, or ability to rapidly produce force in the activities described above, during the single leg drop vertical jump and triple hop for distance in ACLR. Birchmeier et al (2019) investigated performance in patients with unilateral ACLR during a single leg drop vertical jump task using RSI. The authors examined associations between RSI and isometric performance with single leg hop and triple hop for distance scores. They reported that 61.8% of variance in triple hop distance was explained by RSI, jump height, and isometric rate of torque development; however, RSI was not found to be associated with single hop distance [23]. The authors suggested that their findings highlighted the importance of using multiple tests, since the single leg hop for distance can determine improvements in knee extension strength but is not related to plyometric activities encountered in sporting tasks [23]. Similarly, Lloyd et al (2020) explored reactive strength ratios (RSR), the ratio of flight time to contact time, during the triple hop for distance using a floor level optical measurement system. Despite reporting that 80% of ACLR patients reached 90% LSI in triple hop distance, the authors found that only 35% and 45%, respectively, achieved a 90% LSI in RSR across the two rebounds considered [24]. Although individual hop distances were not reported, given the instructions to control the final landing in the triple hop, one might expect different strategies between hops would influence the RSR as shown by the difference in flight times between hops one and two [24]. Therefore, it may be important to consider RSR in other commonly used hop tests that target an athlete’s ability to rapidly accelerate and reach top speed.

The six-meter timed hop (6mTH) provides another opportunity to evaluate RSR, despite its low sensitivity to detect continued functional deficits [25–28]. Nevertheless, interlimb symmetry on single leg hop and 6mTH tests is associated with isokinetic peak torque at low velocities (60 deg∙s^-1^), but not high velocities (300 deg∙s^-1^) [29], which is counterintuitive given the high joint angular velocities observed during single leg loading tasks [30]. Recent research findings connecting the single leg hop and strength may provide a basis for reassessing the associations of the 6mTH and isokinetic strength [23]. Repurposing this test to provide a measure of lower extremity stretch-shortening cycle capacity may improve its role in RTS testing batteries to augment other functional tests, potentially in the absence of available isokinetic strength testing.

Therefore, the purpose of this work was to provide a simple method for determining RSR during the 6mTH test and evaluate the association between RSR and isokinetic peak torque in ACLR patients. Recognizing that different strategies can be used to complete the test (small versus large hops), we used 6mTH speed as a correction factor for RSR to mitigate effects of hopping strategies. We hypothesized that after adjusting for speed to control for 6mTH performance, RSR would show a strong positive association with isokinetic peak torque, particularly when higher isokinetic speeds were employed. Additionally, we sought to assess whether RSR would be consistent with interlimb isokinetic peak torque deficits, but given mixed evidence in RSI across sex, would be resistant to differences in these groups. We hypothesized that the speed adjusted RSR would not show sex differences, therefore making it a stable measure as compared to isokinetic testing.

## Materials and methods

### Participants

This study included data from 29 patients that underwent ACLR at least four months prior to testing. Participants were recruited between May 2023 and December 2023. All ACLR procedures were performed by two board-certified sports medicine orthopedic surgeons at a university medical center that performs over 350 ACLR surgeries per year. Inclusion criteria were that participants were at least 13 years of age, had ACLR performed by one of the two surgeons, and were cleared by their surgeon to complete hop testing. Prospective participants were excluded from the study if they had any post-operative range of motion or weight-bearing restrictions or were not medically cleared to complete hop testing.

This study was part of a larger ACLR point of care project and was approved by the University’s Institutional Review Board for Human Subjects Research (ID: 20230400) and written informed consent was obtained from each participant. For participants under 18 years of age, written informed consent was obtained from both the participant and their guardian prior to testing.

### Testing procedures

Participants’ height and mass were assessed upon arrival at the laboratory. A Biodex System 4 Isokinetic Dynamometer (Biodex Medical Systems, Shirley, NY) was used to assess peak torque during a concentric-concentric knee extension and flexion test [31]. Participants were positioned on the Biodex by aligning the axle of the powerhead with the lateral femoral epicondyle. Restraining straps were placed across the chest, waist, and thigh to isolate them from the other musculature as much as possible. Participants were instructed to place their hands across their chest for the duration of testing. Proper familiarization with the machine was provided, and all patients completed a warm-up set, followed by three testing sets. The warm-up was performed at a speed of 270 deg·s^-1^ for 10 repetitions. After the warm-up set, all patients were given a two-minute rest period before beginning the testing sets. After the rest period, participants completed three sets of isokinetic knee extension at 60, 180, and 300 deg·s^-1^. Each set consisted of three repetitions, and a two-minute rest period was given between testing sets. The starting side (operated, non-operated) for testing was determined in a randomized order for each participant, and vocal encouragement was standardized by instructing participants to perform each testing repetition as fast and forcefully as possible [32]. Isokinetic peak torque was collected for each set from the Biodex machine.

Following isokinetic testing, participants were seated on an exam table while thirty-five reflective markers were placed on the trunk and lower body using a modified Plug-in Gait protocol. Markers were placed bilaterally on the anterior and posterior superior iliac spines, iliac crests, anterior and lateral thighs, medial and lateral femoral epicondyles, fibular heads, tibial tuberosities, medial and lateral malleoli, posterior calcaneus, and the 2^nd^ and 5^th^ proximal metatarsophalangeal joints [33]. Additional markers were placed on the manubrium just below the suprasternal notch, xiphoid process, 7^th^ cervical vertebrae, 10^th^ thoracic vertebrae, medial border of the right scapula, and lateral to the acromioclavicular joint (positioned bilaterally).

This study used a 10-camera optoelectronic motion capture system (Qualisys AB, Gothenburg, Sweden) to track participants during hop testing at a recording rate of 100 frames per second. Participants were first positioned in the center of the motion capture space for an initialization trial that was used to augment marker labeling and initialize the eight-segment skeletal model during post-processing. Following the static trials, participants completed a series of hop tests which were ordered using a random number generator. Only the 6mTH was used in the present study, therefore only the protocol for this test is outlined below.

The 6mTH tests were completed on each side, and the starting limb was randomized prior to the testing session. Prior to each test, two practice trials were given for each limb. Following practice attempts, patients completed all testing trials on the starting limb followed by testing the contralateral limb. At least three trials were completed on each side and additional trials were given if minimal detectable change values for the 6mTH test were exceeded [34]. The average of the three best trials was used in the statistical analyses. Participants were instructed to hop on one leg as fast as possible from the starting line through two cones placed six meters away and then gradually slow down to a stop, after which they were instructed to walk at a comfortable pace back to the starting line. The instructions to start the test were given by the researcher running the motion capture system to control the time between trials. After the trial was started, the motion capture system recorded continuously for 15 seconds. The 6mTH time was recorded using a stopwatch by one researcher who was instructed to start the time at the moment of the participant’s first heel lift and end the time at the point the participant crossed the six-meter finish line [35].

### Data analysis

After completion of the hop testing session, marker processing was completed in the Qualisys Track Manager software (Qualisys AB, Gothenburg, Sweeden) using a custom trained Automatic Identification of Markers (AIM) model. First, all marker discontinuities were resolved in the initialization trial and the trial was added to the AIM model. Next, all 6mTH testing trials were retracked using the updated AIM model and cropped to regions of interest. Given that the 6mTH course exceeds the volume of the motion capture system, the trials were cropped from a point prior to the participant crossing a line two meters away from the motion capture origin and to the point at which they crossed the finish line and landed. Biomechanical data were analyzed during the final four meters of the hop test. All marker discontinuities were resolved within this region using rigid-body and cubic spline filling. Following marker processing, trials were exported to Visual 3D (C-motion Inc., Germantown, MD) for skeletal modeling.

A static portion of the initialization trial was used to build the eight-segment trunk and lower body model. No model constraints were used; therefore, all segments assumed three linear and three rotational degrees of freedom. All marker trajectories used to drive the model were lowpass filtered using a 2^nd^ order Butterworth filter with a cutoff frequency of 15Hz [36]. Joint and segment kinematics were calculated within Visual 3D and were consistent with the right-hand rule, where positive rotation around a joint axis is in the direction of the finger curl with the thumb pointed along the axis. This was ensured for segments by recomputing each pose relative to a virtual laboratory coordinate system which was consistent with the direction of hop test progression. The sagittal knee angle was inverted to maintain consistent interpretation with other lower extremity joints. To maintain consistency between right and left sides, the frontal and transverse joint angles were negated on the left side.

**Fig 1:**
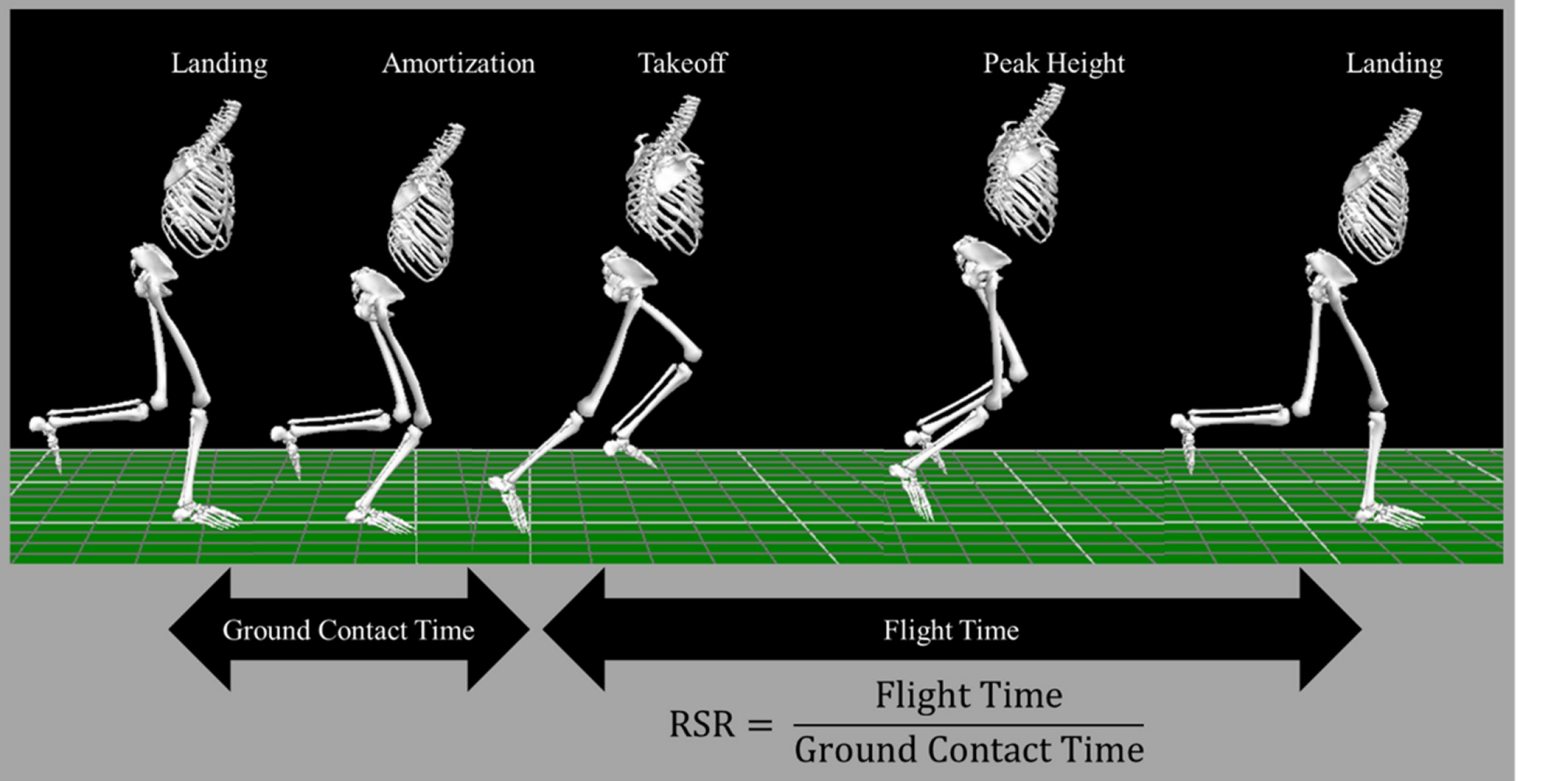
Example of timed hop. Left to right: landing, zero velocity of pelvis (amortization), takeoff, peak height, and landing. RSR: Reactive Strength Ratio.

Given that a considerable number of force platforms would be needed to determine landing and takeoff events to calculate RSR in the triple hop or 6mTH, prior studies have used floor level optical measurement systems or manual identification in videos [24, 37]. Recognizing the need to reduce equipment barriers, remove human error, and build accessible methods across motion capture methods, to calculate RSR during the 6mTH in the present study, landing and takeoff events were determined in the region of interest using the pelvis center of mass vertical velocity (Fig 1) [38]. In this method, the local maximum vertical velocity of the pelvis corresponded with takeoff events, whereas localized minimums corresponded with landings. Consecutive events, landing-takeoff and takeoff-landing were used to determine ground contact and flight times. RSR was calculated as the ratio of flight time to ground contact time.

### Statistical analysis

Mean values and standard deviations were computed across three repetitions for relative peak torque at each isokinetic speed and across three trials for 6mTH time and RSR. Noting the different strategies available to complete the 6mTH, RSR was adjusted for speed (RSR_Adj_) (determined from 6mTH time), whereas smaller hops resulting in small displacements in the horizontal direction (typically showing higher RSR) would be penalized. Repeated measures correlations were used to determine associations between isokinetic and hop testing variables (timed hop time, RSR, RSR_Adj_) [39–41]. Repeated measures correlations account for non-independence of data points taken from the same subject, which would not satisfy the assumptions for linear correlation approaches [39]. Correlation coefficients were interpreted as very high (.90–1.00), high (.70-.90), moderate (.50-.70), low (.30-.50), and negligible (.00-.30) [42].

Leg and sex differences for isokinetic (relative peak torque in Nm∙kg^-1^ at 60, 180, and 300 deg∙s^-1^) and hop testing (timed hop time, RSR, and RSR_Adj_) variables were explored using a mixed model ANOVA. If significant main or interaction effects were noted, least significant difference (LSD) post-hoc tests were used to determine the source. Repeated measures correlations were conducted in R Statistical Software (Version 4.3.2, R Core Team 2021) using the *rmcorr* package [39]. Group comparisons were conducted using SPSS (Version 29, IBM Corp., Armonk, NY). The *a-priori* level of significance was set at .05.

During our *a-priori* power analysis using G*Power (Version 3.1) [43, 44], we calculated a total sample size of N = 20 for our correlation approach, using a one tail test, power of .95, and a medium effect size (*r* = .609) taken from a previously reported association between RSI and isometric peak torque [23]. For the *a-priori* power analysis for group comparisons we calculated a total sample size of N = 36 using a repeated measures within-between interaction model, power of .95, with two groups (sex) and four measurements (leg by sex), and a medium effect size of .25 taken from results of normalized quadriceps’ isokinetic peak torque at 60 deg∙s^-1^ reported by Roman et al. 2021 [45].

Intrasession reliability of RSR was examined using a two-way mixed effects model with an average of *k* measurements (ICC[3, *k*]) to determine absolute agreement between the repeated 6mTH trials. The ICC estimate was interpreted using the guidelines from Koo and Li, 2016 [46], and indicated good to excellent intrasession reliability (ICC = 0.878; 95% Confidence Interval: 0.800, 0.929) in RSR, with a standard error of the measurement equal to 0.138 and minimum detectable change score of 0.382.

### Results and discussion

Patient characteristics are presented in Table 1.

**Table 1:** Descriptive characteristics of the participants.

	Overall (N = 29)	Male (N = 17)	Female (N = 12)
	Mean ± SD	Mean ± SD	Mean ± SD
Time Since Surgery (weeks)	40.97 ± 15.85	35.76 ± 12.04	48.33 ± 18.09
Mass (kg)	71.90 ± 14.69	80.52 ± 13.17	59.70 ± 4.38
Height (m)	1.70 ± 0.08	1.76 ± 0.06	1.63 ± 0.04
Age (years)	26.03 ± 8.99	26.29 ± 7.93	25.67 ± 10.68

SD: Standard Deviation.

### Association between hop testing and isokinetic peak torque

**Fig 2:**
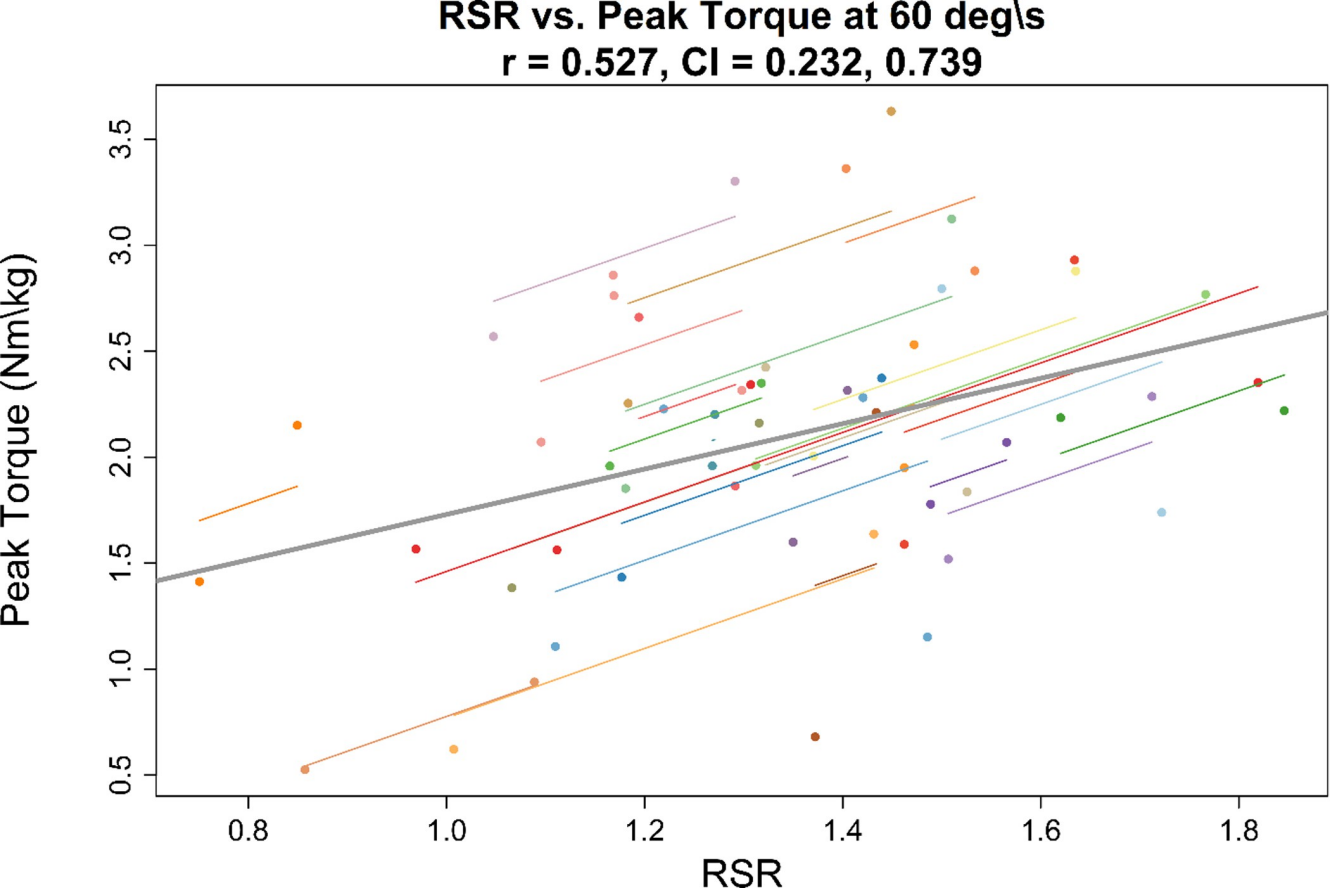
Repeated measures correlation. Repeated measures correlation between reactive strength ratio (RSR) and relative isokinetic peak torque at 60 deg∙s^-1^ expressed in Nm∙kg^-1^. The gray line indicates the overall line of fit and individual lines are shown for each participant. The correlation coefficient (r) and confidence interval (CI) are shown in the plot title.

**Fig 3:**
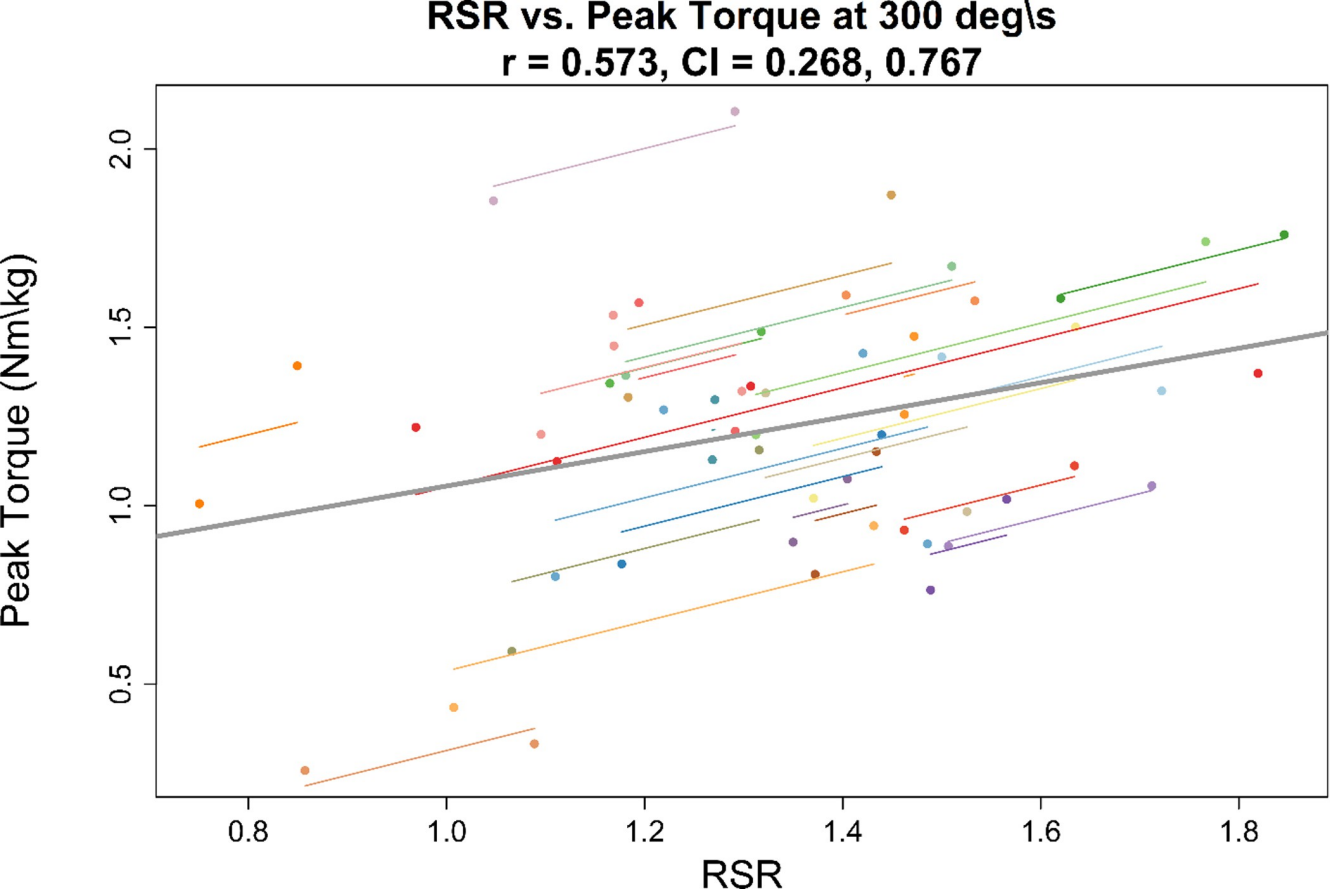
Repeated measures correlation. Repeated measures correlation between reactive strength ratio (RSR) and relative isokinetic peak torque at 300 deg∙s^-1^ expressed in Nm∙kg^-1^. The gray line indicates the overall line of fit and individual lines are shown for each participant. The correlation coefficient (r) and confidence interval (CI) are shown in the plot title.

Summary results for correlational analyses are shown in Table 2 (correlations with isokinetic average power are shown in S1 Table). An example of the association between RSR and isokinetic peak torque is shown in Figs 2 and 3. Correlations between timed hop time and relative isokinetic peak torque were low with confidence intervals showing low to moderate negative association between variables across speeds. Correlations showed moderate positive associations between all RSR variables and relative isokinetic peak torques at all speeds. Confidence intervals showed a range of negligible/low association to high associations between isokinetic and RSR variables.

**Table 2:** Repeated measures correlations between reactive strength and relative quadriceps’ isokinetic peak torque.

	60 deg∙s^-1^		180 deg∙s^-1^		300 deg∙s^-1^	
	*r*	CI	*r*	CI	*r*	CI
Time	-0.407	-0.578, -0.106	-0.415	-0.615, -0.019	-0.392	-0.579, 0.015
RSR	0.527	0.255, 0.740	0.545	0.242, 0.739	0.573	0.268, 0.767
RSR_Adj_	0.577	0.333, 0.759	0.563	0.291, 0.736	0.554	0.297, 0.736

*r*: Correlation coefficient, CI: 95% Confidence Interval, Time: Timed hop time in seconds, RSR: Reactive Strength Ratio, RSR_Adj_: RSR adjusted for speed of performance.

### Leg and sex differences

Descriptives for isokinetic and hop testing variables are shown in Table 3. Results of the mixed model ANOVA and post-hoc comparisons are shown in Table 4 and in S2 Table. Significant main effects for leg and sex were found, but no significant interaction effects were noted. Differences by leg were noted for relative isokinetic peak torque at 60 deg∙s^-1^, 180 deg∙s^-1^, and 300 deg∙s^-1^, timed hop time, RSR, and RSR_Adj_. Differences by sex were noted for relative isokinetic peak torque at all speeds and for timed hop time.

**Table 3:** Means and standard deviations for isokinetic and hop testing variables by leg and sex. Isokinetic peak torques are expressed in Newton-meters relative to body mass in kilograms (Nm∙kg^-1^).

	Overall (N = 29)		Male (N = 17)		Female (N = 12)	
	OP	Non-Op	OP	Non-Op	OP	Non-Op
PT60	1.74 ± 0.60	2.45 ± 0.515	1.97 ± 0.61	2.58 ± 0.61	1.40 ± 0.43	2.27 ± 0.26
PT180	1.35 ± 0.46	1.73 ± 0.37	1.54 ± 0.47	1.85 ± 0.41	1.09 ± 0.30	1.57 ± 0.25
PT300	1.09 ± 0.37	1.35 ± 0.32	1.22 ± 0.40	1.44 ± 0.36	0.90 ± 0.21	1.24 ± 0.22
Time (s)	2.29 ± 0.73	2.17 ± 0.63	2.01 ± 0.40	1.93 ± 0.36	2.69 ± 0.91	2.53 ± 0.76
RSR	1.28 ± 0.23	1.40 ± 0.23	1.27 ± 0.25	1.35 ± 0.25	1.28 ± 0.20	1.49 ± 0.19
RSR_Adj_	3.56 ± 0.97	4.09 ± 1.08	3.89 ± 0.91	4.26 ± 0.86	3.10 ± 0.89	3.84 ± 1.33

Op: Operative leg, Non-Op: Non-Operative leg, PT60: Relative peak torque at 60 deg∙s^-1^, PT180: Relative peak torque at 180 deg∙s^-1^, PT300: Relative peak torque at 300 deg∙s^-1^, Time: Timed hop time, RSR: Reactive Strength Ratio, RSR_Adj_: RSR adjusted for speed of performance.

**Table 4:** ANOVA results for main effects. Isokinetic peak torque is expressed in Nm∙kg^-1^.

	F(1,27)	p	Partial η
Operative vs. Non-Operative			
PT60	69.759	< .001	0.721
PT180	62.647	< .001	0.699
PT300	53.291	< .001	0.664
Time	5.154	< .05	0.160
RSR	10.436	< .01	0.279
RSRadj	12.574	< .01	0.318
Male vs. Female			
PT60	6.245	< .05	0.188
PT180	7.409	< .05	0.215
PT300	4.596	< .05	0.145
Time	8.24	< .01	0.234
RSR	1.044	0.316	0.037
RSRadj	3.242	0.083	0.107
Interaction			
PT60	2.164	0.153	0.074
PT180	3.164	0.087	0.105
PT300	2.748	0.109	0.092
Time	0.563	0.46	0.02
RSR	2.28	0.143	0.078
RSRadj	1.433	0.242	0.05

PT60: Relative peak torque at 60 deg∙s^-1^, PT180: Relative peak torque at 180 deg∙s^-1^, PT300: Relative peak torque at 300 deg∙s^-1^, Time: Timed hop time, RSR: Reactive Strength Ratio, RSR_Adj_: RSR adjusted for speed of performance.

For isokinetic peak torque, compared to the non-operative leg, patients showed strength deficits in the operative leg at 60 deg∙s^-1^ (MD ± SE: -0.74 ± 0.09 Nm∙kg^-1^, p < .001, d = -1.28), 180 deg∙s^-1^ (MD ± SE: -0.39 ± 0.05 Nm∙kg^-1^, p < .001, d = -0.91), and 300 deg∙s^-1^ (MD ± SE: -0.28 ± 0.04 Nm∙kg^-1^, p < .001, d = -0.77). Additionally, patients showed slower (longer) timed hop times (MD ± SE: 0.13 ± 0.06 s, p < .001, d = 0.18) in the operative compared to the non-operative leg as well as lower RSR (MD ± SE: -0.14 ± 0.04, p < .001, d = -0.55) and RSR_Adj_ (MD ± SE: -0.56 ± 0.16, p < .001, d = -0.51).

For sex differences, female patients showed lower isokinetic strength compared to male patients at 60 deg∙s^-1^ (MD ± SE: -0.44 ± 0.18 Nm∙kg^-1^, p < .05, d = -0.94), 180 deg∙s^-1^ (MD ± SE: -0.37 ± 0.13 Nm∙kg^-1^, p < .05, d = -1.03), and 300 deg∙s^-1^ (MD ± SE: -0.25 ± 0.12 Nm∙kg^-1^, p < .05, d = -0.81). Female patients also showed slower (longer) timed hop times (MD ± SE: 0.64 ± 0.22 s, p < .05, d = 1.08) compared to male patients, but no differences in RSR and RSR_Adj_ were detected between groups.

## Discussion

The purpose of this work was to provide a method to determine RSR during the 6mTH test and evaluate its association with isokinetic peak torque in ACLR patients. The hypothesis that RSR_Adj_ would show a strong positive association with isokinetic peak torque, particularly with the higher isokinetic speeds, was not supported by the current results. Additionally, the expectation that RSR_Adj_ would provide higher associations with isokinetic variables than RSR was not supported, as the raw RSR showed moderate positive associations across speeds. Important to this work, the original 6mTH performance metric–time–showed low associations with isokinetic peak torque, yet the purpose of its measurement could be preserved in the speed adjustment for RSR, albeit some modifications are necessary in future work.

The association between RSR and isokinetic peak torque did not increase with higher isokinetic speeds as originally thought. While others have documented high knee joint angular velocities during single leg loading tasks [30], different landing and takeoff strategies during the 6mTH may mask this effect and are not considered in the speed correction for RSR. Others have found that a similar performance measure, RSI, is influenced by landing strategies in the drop vertical jump, with softer landing techniques showing lower RSI compared to self-selected or stiff landing strategies [47]. These authors also concluded that without notable differences in the knee valgus angles across landing strategies, soft landing strategies pose a larger problem for performance than the tradeoff for ACL injury risk reduction [47]. At this point, differences in coordination strategies among lower extremity joints during the 6mTH, particularly the contribution of the knee joint, may have a larger impact on RSR than we originally thought and may be important to expand upon in future work using lower extremity kinematic information.

Although prior research has shown associations between maximum- and reactive-strength, differences between concentric-concentric isokinetic testing and the eccentric-concentric nature of the 6mTH may explain why higher associations were not found with increasing isokinetic speeds. Additional considerations of eccentric to concentric ratios during 6mTH testing may also be important to include when assessing performance relative to concentric-concentric isokinetic testing. In fact, techniques from countermovement jump testing with force platforms can be replicated to achieve this [48], using the zero-crossing of the pelvis vertical velocity to demarcate eccentric and concentric phases.

This study attempted to provide a simple method of determining stretch-shortening cycle capacity in the 6mTH to improve availability of objective methods and frequency of testing [1] and to address the failure of traditional outcome measures (timed hop time) to detect continued functional deficits in ACLR [25–28]. While the equipment involved in this project may be unavailable to many clinicians, the fundamentals are relatively simplistic relying only on pelvis linear velocity and may be applied to other open-source motion capture systems or inertial sensors. Current results partially support our second hypothesis as raw and adjusted RSR were able to detect interlimb differences in the cohort of ACLR patients tested in this study with medium effect sizes. Acknowledging that large interlimb effects were noted across isokinetic variables, only a small effect was found for timed hop time, which may suggest RSR could viably replace this metric in standard 6mTH testing if accessible biomechanical methods can be developed. Along these lines, raw RSR may be preferred to its speed-adjusted counterpart, as shortcomings in the 6mTH performance strategies could have influenced results with for the RSR_Adj_ [35]. Correlation results support this notion as only small differences in coefficients were observed between RSR and RSR_Adj_ in the current work.

Prior research, and the current work, collectively show that isolated isometric and isokinetic testing differ across age, sex, and graft conditions following ACLR [45, 49], which reduces the viability of normative data, leaving clinicians and patients with limited options to determine progress. Considerable efforts have been made to provide reference values, yet large gaps remain in many subgroups due the large number of independent factors across ACLR and rehabilitation protocols [50]. The current work aimed to provide an alternative metric that would be generalizable across groups while maintaining the ability to determine interlimb deficits. While large effects were noted in isokinetic strength between male and female patients, the hypothesis that RSR would not show sex differences is only partially supported due to the current sampling approach. While comparisons between male and female participants were not statistically significant, inconsistencies in effect size magnitude and direction between RSR and RSR_Adj_ limited further interpretation at this point. Others have found that RSI (in countermovement jumps) is lower in female participants when adjusting for lower extremity isometric strength and rate of force development [21]. Similarly, across collegiate sports, male athletes have shown higher RSIs, but statistically significant differences could be sport dependent [21, 22]. Given the heterogenous group of participants in the present study, no attempts were made to control for sport type, level of participation, and age; which may have weakened the capacity of RSR to discriminate sex differences. Further methodological development to improve the application of instrumented hop testing may help answer the generalizability of this approach in determining functional recovery after ACLR.

### Limitations

This study had several limitations which may have affected our results. First, all patients were examined at least four months after surgery and with clearance from their treating physician, yet consistent time points were not examined. This could have largely influenced between group results, as males were tested at an average of nine months versus 11 months post-op in the female group. Second, small errors in the landing and takeoff detection method could have influenced RSR results, although RSR measures were higher than values reported in ACLR during the triple hop for distance [24], and similar to values reported in healthy male volleyball players performing the triple hop for distance [37]. Third, the use of hand timing in the 6mTH test likely influenced the results across groups as well as the validity of the adjusted RSR, therefore other equipment like timing gates and light systems (e.g., FITLIGHT) may be needed in future work. Finally, only isokinetic, timed hop time, and RSR variables were considered; while the effect of different movement strategies was not explored. Secondary analyses are warranted to determine the potential effect of lower extremity movement patterns on RSR performance.

## Conclusions

Measures of lower extremity stretch-shortening cycle capacity were explored during the 6mTH as potential alternatives to isokinetic testing. Moderate associations were found between RSR and isokinetic peak torque while all variables showed significant interlimb differences. Isokinetic peak torque differed across sex, but limited evidence was found for RSR metrics. Preliminary results are promising but further development is needed to validate other accessible technologies capable of making these measurements. Pending these developments, the effects of movement strategies, demographics, and levels of participation on RSR can then be explored.

## Supporting information

S1 Table Repeated measures correlations between reactive strength and relative quadriceps’ isokinetic average power across speeds. (DOCX)

S2 Table Mean comparisons across leg and sex. Isokinetic peak torque is expressed in Nm∙kg^-1^.
(DOCX)

S1 Data (XLSX)
