# Supplementary material for: A hop testing alternative for functional performance following anterior cruciate ligament reconstruction
Source: PLoS One. 2024 Aug 16;19(8):e0309003. doi: 10.1371/journal.pone.0309003 (PMC11329148; doi:10.1371/journal.pone.0309003)
Supplement: S1 Table — (DOCX) [file pone.0309003.s001.docx]

**S1 Table.** Repeated measures correlations between reactive strength and relative quadriceps’ isokinetic average power across speeds.

|  | 60 deg∙s^-1^ | | 180 deg∙s^-1^ | | 300 deg∙s^-1^ | |
| --- | --- | --- | --- | --- | --- | --- |
|  | *r* | CI | *r* | CI | *r* | CI |
| RSR | 0.360 | 0.006, 0.623 | 0.365 | -0.044, 0.649 | 0.358 | -0.039, 0.655 |
| RSR_Adj_ | 0.426 | 0.084, 0.695 | 0.437 | 0.084, 0.711 | 0.392 | 0.018, 0.654 |

RSR: Reactive Strength Ratio; RSR_Adj_: Adjusted RSR.
