# Supplementary material for: A hop testing alternative for functional performance following anterior cruciate ligament reconstruction
Source: PLoS One. 2024 Aug 16;19(8):e0309003. doi: 10.1371/journal.pone.0309003 (PMC11329148; doi:10.1371/journal.pone.0309003)
Supplement: S2 Table — Isokinetic peak torque is expressed in Nm∙kg-1. (DOCX) [file pone.0309003.s002.docx]

**S2 Table.** Mean comparisons across leg and sex. Isokinetic peak torque is expressed in Nm∙kg^-1^.

|  | MD ± SE | CI | p | d |
| --- | --- | --- | --- | --- |
| Operative vs. Non-Operative | | | | |
| PT60 | -0.74 ± 0.09 | -0.92, -0.56 | <.001 | -1.28 |
| PT180 | -0.39 ± 0.05 | -0.50, -0.29 | <.001 | -0.91 |
| PT300 | -0.28 ± 0.04 | -0.35, -0.20 | <.001 | -0.77 |
| Time (s) | 0.13 ± 0.06 | 0.01, 0.24 | <.001 | 0.18 |
| RSR | -0.14 ± 0.04 | -0.23, -0.05 | <.001 | -0.55 |
| RSR_Adj_ | -0.56 ± 0.16 | -0.88, -0.24 | <.001 | -0.51 |
| Female vs. Male | | | | |
| PT60 | -0.44 ± 0.18 | -0.80, -0.08 | <.05 | -0.94 |
| PT180 | -0.37 ± 0.13 | -0.64, -0.09 | <.05 | -1.03 |
| PT300 | -0.25 ± 0.12 | -0.49, -0.01 | <.05 | -0.81 |
| Time (s) | 0.64 ± 0.22 | 0.18, 1.10 | <.05 | 1.08 |
| RSR | 0.08 ± 0.08 | -0.08, 0.23 | .316 | 0.38 |
| RSR_Adj_ | -0.61 ± 0.34 | -1.31, 0.09 | .083 | -0.68 |

MD: Mean Difference, SE: Standard error of the difference, CI: Confidence Interval, d: Effect size, PT60: Relative peak torque at 60 deg∙s^-1^, PT180: Relative peak torque at 180 deg∙s^-1^, PT300: Relative peak torque at 300 deg∙s^-1^, Time: Timed hop time, RSR: Reactive Strength Ratio, RSR_Adj_: Adjusted RSR.
